# Phase-matched second harmonic generation with on-chip GaN-on-Si microdisks

**DOI:** 10.1038/srep34191

**Published:** 2016-09-30

**Authors:** I. Roland, M. Gromovyi, Y. Zeng, M. El Kurdi, S. Sauvage, C. Brimont, T. Guillet, B. Gayral, F. Semond, J. Y. Duboz, M. de Micheli, X. Checoury, P. Boucaud

**Affiliations:** 1 Centre de Nanosciences et de Nanotechnologies, CNRS, Univ. Paris-Sud, Université Paris-Saclay, Bâtiment 220, Rue André Ampère, F-91405 Orsay, France; 2 CRHEA-CNRS, Rue Bernard Grégory, F-06560 Valbonne, France; 3 Laboratoire Charles Coulomb (L2C), UMR 5221, CNRS-Université de Montpellier, F-34905 Montpellier, France; 4 Univ. Grenoble Alpes, F-38000 Grenoble, France; 5 CEA, INAC-PHELIQS, Nanophysique et semiconducteurs group, F-38000 Grenoble, France; 6 Laboratoire de Physique de la Matière Condensée, UMR CNRS 7336, Université de Nice-Sophia Antipolis, 06108 Nice, France

## Abstract

We demonstrate phase-matched second harmonic generation in gallium nitride on silicon microdisks. The microdisks are integrated with side-coupling bus waveguides in a two-dimensional photonic circuit. The second harmonic generation is excited with a continuous wave laser in the telecom band. By fabricating a series of microdisks with diameters varying by steps of 8 nm, we obtain a tuning of the whispering gallery mode resonances for the fundamental and harmonic waves. Phase matching is obtained when both resonances are matched with modes satisfying the conservation of orbital momentum, which leads to a pronounced enhancement of frequency conversion.

The development of on-chip optical circuits is generating numerous progress in optical interconnects, sensing and quantum technologies. The advantage of on-chip optical circuits is the capacity to integrate on a small footprint active and passive devices. One of the driving forces of their development is the integration of different types of microresonators exhibiting high quality factors and small modal volumes. Both features lead to an enhancement of nonlinear interactions that can be exploited for broadband light generation, optical switches and transfer of coherent excitation to shorter or longer wavelengths^1^,^2^. A particularly useful nonlinear interaction is the one associated with the second order nonlinear susceptibility. The second order nonlinearity can lead to Pockel’s effect, harmonic generation, frequency mixing or spontaneous down-conversion. With a noncentrosymmetric material that exhibits a non-zero second order nonlinear susceptibility, one can either transfer a coherent excitation to shorter wavelength or generate photons from the visible to the telecom or infrared band.

One of the key challenges for on-chip optical circuits is the combination of materials with specific properties and their transparency spectral range. Silicon is a material of choice for near-infrared but is strongly absorbing at wavelengths shorter than 1 *μ*m thus ruling out its use in the visible. GaAs is also absorbing at wavelengths shorter than 870 nm and harmonic experiments require an optical excitation around 1.75–2 *μ*m to avoid absorption^3^,^4^. Lithium niobate exhibits very high second order nonlinear coefficients but does not provide integrated emitters^5^,^6^. SiN is an interesting platform but the material is centrosymmetric, i.e. no second order processes are allowed except for surface effects^7^, and there are no efficient emitters as well. The III-nitride materials are on the contrary very attractive for the development of on-chip advanced optical circuits. The III-nitride materials are the materials of reference for visible and UV emission and as opposed to silicon can provide efficient light emitting diodes or lasers. The wide transparency range of the III-nitride materials is a key asset for nonlinear mixing, i.e. frequency doubling and down conversion, can occur either in the visible or in the telecom band. The III-nitride materials can be grown on silicon or silicon-on-insulator substrates^8^,^9^, thus offering a low-cost platform for the development of monolithic on-chip optical circuits.

In the literature, several optical functions have been reported with III-nitrides deposited by sputtering on oxide including harmonic generation and opto-mechanical devices^10^,^11^,^12^. High quality factors (400000) have been reported in large diameter microrings with polycrystalline aluminum nitride films^13^. GaN-on-silicon bonding technology was also demonstrated for on-chip optical interconnects^14^. A bonding technology was used in ref. 15 to fabricate crystalline GaN-on-oxide on silicon and to achieve resonant second harmonic generation in 80 *μ*m diameters microrings. GaN directly grown on silicon is also attracting a significant interest. Several types of microresonators were reported with this platform, either two-dimensional photonic crystals^16^,^17^,^18^,^19^,^20^ or microdisks^9^,^21^. We have recently reported on a III-nitride photonic platform with microdisk resonators optically addressed with bus waveguides^22^. This microdisk platform is well adapted for harmonic generation using wavelength-scale compact microresonators with a diameter smaller than 10 *μ*m.

One of the key issue for harmonic generation is the achievement of phase matching that can lead to an efficient energy transfer provided that the mode overlap between pump and harmonic is sufficiently high. In this letter, we report on phase-matched second harmonic generation in doubly resonant GaN microdisks on silicon. The phase matching is demonstrated by tuning the disk diameter by steps of 8 nm in order to reach the double resonance condition and conservation of orbital momentum. The systematic variation of one microdisk parameter (i.e. the diameter) allows one to track the resonant harmonic mode and the occurrence of doubly-resonant second harmonic generation for a specific diameter. It provides a direct evidence of enhanced conversion efficiency when phase matching occurs. In the literature, other reports of phase matching with microdisks have been obtained using a limited number of samples^4^, thermo-optic phase matching^23^ and a combination of temperature and stress^24^. The demonstration of phase matching conversion with microdisks is an important step to demonstrate the full potential of III-nitride optical circuits on silicon.

## Results

Different strategies are available for phase matching. The classical one is the use of birefringent materials to compensate for the index difference between pump and harmonic. Quasi-phase matching can be obtained by using domain inversions that reverse periodically the sign of the nonlinear susceptibility^25^. In microdisk resonators with cylindrical symmetry, phase matching depends on the symmetry of the nonlinear susceptibility that leads to specific selection rules. In materials with second order diagonal elements, i.e. like 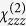, phase matching can be obtained by compensating material dispersion through modal dispersion. In materials like arsenides with a zinc-blende crystal symmetry, the non diagonal second order susceptibility element 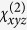 changes its sign following a rotation of 90°. 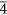 quasi-phase matching with materials with 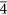 symmetry becomes possible without the artificial creation of periodic domains^4^,^26^. Other configurations are also available depending on the symmetry of the nonlinear tensor^23^. Cyclic birefringent phase matching has been demonstrated in BBO disks^27^ as well as modal phase matching for third harmonic generation^28^. The nitride materials grown on silicon have a wurtzite symmetry and the only second-order nonlinear susceptibilities are 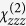, 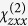, 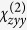 with symmetry in the permutation of the last two indices^29^. In this work, we demonstrate phase matched harmonic generation mediated by the 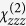 susceptibility element between two TM-polarized (E//z) whispering gallery modes resonant at the fundamental and harmonic wavelengths. The modes confined in the microdisks can be labeled through vertical, radial and azimuthal (l, n, m) indices, l and n representing the number of nodes in the vertical and radial spatial profiles and 2 m the number of antinodes in the azimuthal near-field profile. With the 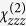 susceptibility, the frequency and phase matching imply conservation of energy and orbital momentum, corresponding to 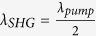 and Δ*m* = *m*_*SHG*_ − 2*m*_*pump*_ = 0. As explained below, we will use the combination of different radial order modes TM(0, 0, 28) for the pump and TM(0, 2, 56) for the harmonic, i.e. modes differing by a factor of two in their azimuthal numbers.

**Figure 1:**
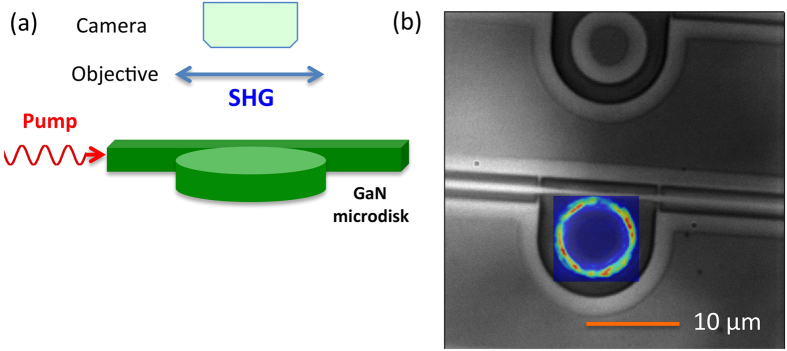
(**a**) Schematics of the experiment. The second harmonic is collected perpendicularly from the layer plane. (**b**) Optical microscopy image of microdisks and its side-coupling bus waveguide. We have superimposed the second harmonic radiated pattern collected from the surface. The harmonic is generated by a resonant whispering gallery mode and appears as a ring at the disk periphery on the image. The harmonic is excited in continuous wave in resonance with the TM(0, 0, 28) mode at 1557 nm (disk diameter 7.92 *μ*m). A filter on the collection path rejects the pump mode.

The experiments were performed using a GaN-on-silicon two-dimensional photonic platform^19^,^22^. The photonic circuit consists of microdisks coupled to free-standing waveguides suspended by nanotethers (See Methods for details). The harmonic experiments were performed with a continuous wave laser source in the telecom band (1500–1630 nm) and the harmonic was collected perpendicularly to the layer plane with a high numerical aperture objective (0.9), as shown schematically in Fig. 1(a). The high numerical aperture objective allows one to collect z-polarized light emitted in directions away from the optical axis^30^. Scattering by the microdisk sidewalls also redirects light towards the vertical direction. Figure 1(b) shows an optical microscope image of a microdisk and its side-coupling bus waveguide. Figure 1(b) also shows the superimposed spatial profile of the second harmonic signal as measured with the camera. The harmonic is observed at the periphery of the microdisk where the whispering gallery modes are located.

### Linear characterization of III-nitride microdisks

**Figure 2:**
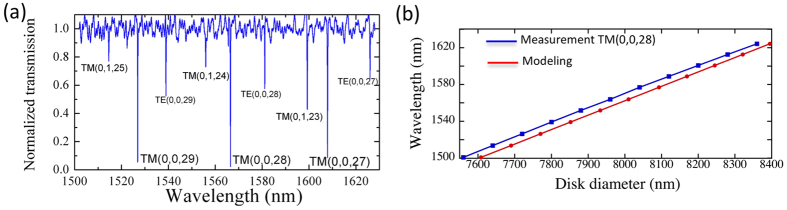
(**a**) Normalized transmission of the photonic circuit for TM-polarized light injection. The modes are labeled according to their vertical, radial and azimuthal indices. The gap distance is 400 nm. (**b**) Spectral dependence of the TM(0, 0, 28) mode as a function of the disk diameter. The blue line corresponds to the measured wavelength as a function of disk diameter coded in the mask. The red line corresponds to the calculated diameter to obtain the same resonance wavelength for the TM(0, 0, 28) mode.

Figure 2(a) shows the transmission spectrum in TM polarization for a microdisk with a nominal 7.997 *μ*m diameter and a nominal thickness of 742 nm. One observes a series of resonant dips that correspond to the coupling to whispering gallery modes. The free spectral range is around 40 nm. The modes have been identified by comparison with modeling using the following index parameters for GaN and AlN at 1550 nm. There is a small birefringence in the material that translates in values of n_0_ = 2.0425 and n_*e*_ =  2.0887 for AlN and n_0_ = 2.2917 and n_*e*_ = 2.3196 for GaN at 1550 nm. The main resonances at 1527, 1567, 1608 nm correspond to the TM(0, 0, 29-28-27) modes. The transmission drop is close to 1, indicating that we are close to the critical coupling with an air gap distance of 400 nm. The loaded quality factors measured for TM-polarized modes vary between 6000 and 13000. These values are lower than those reported in ref. 22 where values up to 80000 were reported for undercoupled waveguides. We attribute the lower value of quality factors to the large thickness of the present structures as compared to the 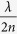 thickness in ref. 22. The larger thicknesses require longer plasma-etching times that lead to a stronger mask erosion and in some cases to an increased sidewall roughness. The bending losses are not dominant for these diameters and sidewall scattering is the dominant source of loss. Let us note that as we seek a double resonance between the fundamental and harmonic resonances, a lower Q relaxes the constraint on the tuning to obtain the resonance condition. Moreover, a lower Q also enhances the bandwidth of frequency conversion. A Q around 10000 is in our case a good compromise between a large enough interaction length while keeping a moderate constraint on the double resonance condition. A very important feature for phase matching is the ability to shift the resonance wavelengths as a function of the disk diameter. This is illustrated in Fig. 2(b) that shows the resonance wavelength of the TM(0, 0, 28) mode as a function of disk diameter. The step variation in diameter for electronic lithography writing is 8 nm, i.e. one thousand times smaller than the disk diameter around 8 *μ*m. Only a fraction of the whole measurements are shown. The measurements are obtained from a series of microdisk-waveguide structures separated by a distance of 20 *μ*m. Figure 2(b) compares the spectral position of the TM(0, 0, 28) mode with the one that has been calculated with an analytical model. The index dispersion used in the calculation is given in the supplementary section. We observe that we can accurately track the mode from 1500 to 1630 nm by changing the disk diameter. One obtains an excellent agreement for the wavelength vs. diameter slope with a standard deviation of 5.5 nm. As compared to the modeling, there is a small offset due to the uncertainty on the refractive indices and on the exact diameter of the processed disk + waveguide. The fabricated disks can be considered smaller than the nominal ones by 48 nm, i.e. 0.6%, for the set of refractive indices considered.

### Second harmonic generation vs. microdisk diameter

**Figure 3:**
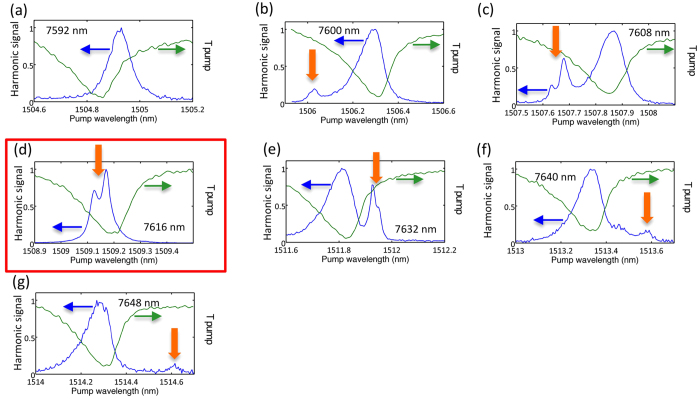
(**a**–**g**) Spectral dependence of the second harmonic signal as a function of the pump wavelength for different disk diameters indicated in the graphs in nm. The green curve corresponds to the pump transmission. The blue curve corresponds to the normalized harmonic signal. The resonant enhancement at the harmonic frequency is indicated by vertical orange arrows. The red square highlights the structure where phase-matched harmonic generation is obtained.

**Figure 4:**
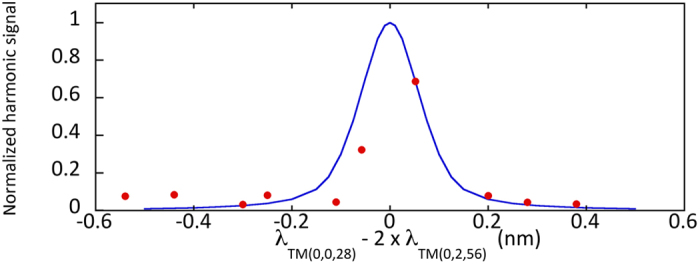
Experimental (dots) and calculated (full line) harmonic signal as a function of the detuning between harmonic and pump. The vertical scale for the experimental data has been adjusted so that the measurement for the 7616 nm diameter disk corresponds to the modeling. The experimental points correspond to data partially shown on Fig. 3.

Achieving double resonant harmonic conversion requires that both pump and harmonic fields are resonant with a whispering gallery mode. To assess this situation, it is possible to perform microdisk spectroscopy around the doubly-resonant signal^5^. In the present experiments, we have investigated the dependence of the harmonic conversion efficiency as a function of the disk diameter. The resonance wavelengths of the TM(0, 0, 28) and TM(0, 2, 56) modes vary as a function of diameter, as shown in Fig. 2(b) for the TM(0, 0, 28) mode, but with different slopes. Consequently, one can expect to find a diameter where both pump and harmonic are resonant with the whispering gallery modes. This occurrence is shown in the supplementary section. The change in the radial index between both modes compensates for the natural dispersion of the microdisk. Note that in the following, phase matching can only occur with the TM(0, 2, 56) mode. Without conservation of the orbital momentum, the double-resonant harmonic signal would be quenched^31^. According to the modeling, only the harmonic mode with a radial index of 2 can lead to phase matching with the TM(0, 0, 28) pump mode. As the loaded quality factors of the modes are around 10000 (full width at half maximum of 0.15 nm), it is required that Δ(*λ*_*TM*(0,0,28)_ − 2*λ*_*TM*(0,2,56)_) ≤ 0.15 nm. We have calculated from the spectral dependence of the resonant modes vs. diameter that 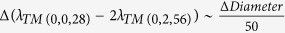. We have thus chosen a 8 nm step of diameter variation, in order to get one resonant diameter within the resonance linewidth. Figure 3 shows the dependence of the normalized second harmonic signal for different disk diameters. The images show the superposition of the transmission in green and the integrated harmonic signal (in blue). The curves have been normalized for clarity, with a different normalization factor for each curve. We will discuss below the spectral dependence of the efficiency that is maximum for the 7616 nm diameter disk (see Fig. 4 and the discussion below). The harmonic amplitude is obtained by spatially integrating the signal as shown in Fig. 1. To perform the experiments, the pump wavelength is adjusted for each diameter in order to be in resonance with the TM(0, 0, 28) mode. The curves shown in Fig. 3 are measured with a weak incident power (6 dBm, i.e. 440 *μ*W in the waveguide close to the microdisk) in order to avoid the nonlinearities associated with residual absorption and the asymmetry of the transmitted signals. The harmonic signal varies however very significantly as a function of the disk diameter. For the 7592 nm diameter, there is only one single resonance as expected when only the pump is resonant with a whispering gallery mode, i.e. no resonant whispering gallery mode at the harmonic frequency. This signal is always present for all diameters as we track the TM(0, 0, 28) mode for each microdisk diameter. A similar signal was also observed for the other TM(0, 0, 29-27) modes when the pump was set in resonance with them. When we increase the diameter, a novel resonance appears on the spectra as underlined by the different vertical orange arrows. We attribute this peak to the resonance of the second order harmonic with a whispering gallery mode, i.e. the wavelength of the harmonic mode is at half of this value. In order to be observed, this mode needs to fall within the spectral range where the second harmonic signal is generated, i.e. within a few linewidth of the fundamental resonant pump mode. The resonance is first observed on the short wavelength part of the spectrum, gets closer to the pump resonance and finally shifts to the long wavelength side of the spectrum. On a limited number of structures (c, d, e), one observes a splitting of the harmonic mode. This splitting could come from a sidewall roughness coupling counter-propagating modes (clockwise and counter-clockwise), thus lifting their spectral degeneracy^32^. The linewidth of the harmonic mode is also reduced as compared to the pump mode (see modeling in the next section). The wavelength difference between the pump fundamental mode TM(0, 0, 28) and two times the wavelength of the harmonic mode TM(0, 2, 56) is minimal for the disk diameter of 7616 nm (0.05 nm as compared to 0.2 nm linewidth). For this disk, the second harmonic signal is dominated by the enhancement due to the double resonance condition of the harmonic. Figure 3 thus highlights all the salient features associated with second harmonic generation: the enhancement of the second harmonic signal with a resonant pump (Fig. 3(a) for example), the enhancement when the harmonic is resonant with a mode ((Fig. 3(b) for example), and the reinforced enhancement when both pump and harmonic satisfying conservation of orbital momentum are resonant and overlap, i.e. phase matching (Fig. 3(d)).

### Evidence of phase-matched second harmonic generation

Following the coupled mode theory^31^,^33^, the circulating second harmonic power |*B*_*SH*_|^2^ can be written as


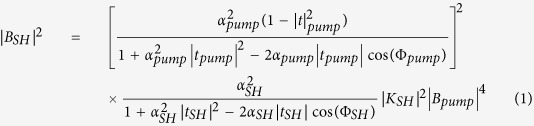


where *α*_*i*_ corresponds to the resonator losses, t_*i*_ to the transmission coefficient for microdisk and waveguide coupling. Both parameters are related to the intrinsic and coupling quality factors by 
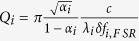
, 
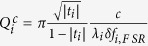
. Φ_*i*_ corresponds to the phase shifts. |*B*_*pump*_|^2^ corresponds to the incident pump power in the waveguide. The spectral dependence of the phase shifts is calculated by linear interpolation of the phase variation as a function of wavelength between modes of the same family differing by their azimuthal number^31^. *δ* *f*_*i*,*F SR*_ is the free spectral range in frequency units. K_*SH*_ accounts for the nonlinear susceptibility and mode overlap^31^.

The first fraction of the right hand side corresponds to the square resonance enhancement with the circulating pump power while the second fraction corresponds to the resonance enhancement of the harmonic. Formula 1 can be used to calculate the spectral dependence of the harmonic response and the conversion efficiency. We deduce from this modeling an average loaded quality factor of 8000 and 15000 for the pump and harmonic respectively corresponding to *α*_*pump*_ = *t*_*pump*_ = 0.9926, and *α*_*SHG*_ = *t*_*SHG*_ = 0.9921, and a free spectral range of 40 nm for the pump. These quality factors are those that best reproduce the linewidth of the harmonic signal in the single and double resonance configuration. Figure 4 shows the spectral dependence of the maximum harmonic signal P_2*ω*_ compared to what is calculated following formula 1. The horizontal axis corresponds to the difference between the pump wavelength and twice the wavelength of the harmonic mode, i.e. the difference between the wavelength pointed by the orange arrow in Fig. 3 and the resonant wavelength of the pump mode corresponding to the dip in the transmission spectrum (right scale in Fig. 3). The modeled harmonic signal has been normalized to one for a zero detuning. One obtains an excellent agreement between the calculated and measured efficiency enhancement. The enhancement is, as expected, very peaked and is a signature of the achievement of phase matching in these GaN-on-Si microdisks. In this situation, within a resonance linewidth, both pump and harmonic are resonant and the orbital momentum is conserved. We note that, while we did scan disk diameters from 7550 to 8300 nm, this large efficiency enhancement was only observed in the spectral range presented in Fig. 4. This demonstration is to our knowledge the first evidence of phase matching with microdisk resonators on an integrated platform obtained by systematically varying a microdisk parameter. We note that if Δ*m* ≠ 0, i.e. the double resonance is obtained with modes that do not satisfy the phase matching condition, there are destructive interferences and one expects to observe a dip in the spectral response as discussed in ref. 31. In the latter case, the conversion efficiency is quenched by orders of magnitude as there is a destructive phase matching and we would not observe the spectral dependence as reported in Fig. 4.

## Discussion

The outside conversion efficiency in mW^−1^ is measured from the ratio between the collected harmonic power and the square of pump power 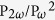 and is estimated as 2 × 10^−9^ mW^−1^ for an incident pump of 1.1 mW in the waveguide close to the microdisk. This value can be compared with other values reported in the literature: 7 × 10^−7^ mW^−1^ in AlGaAs microdisks^34^, 4 × 10^−4^ mW^−1^ in GaP microdisks^23^, 5 × 10^−5^ mW^−1^ in GaAs microdisks^4^ or even 9 × 10^−2^ mW^−1^ in very high quality factor lithium niobate mm-size microdisks and 0.03 mW coupled pump power^5^. The conversion efficiency that we have measured remains limited in these experiments for several reasons. The nonlinear susceptibility of the III-nitride layer is significantly smaller than the one of GaAs (around 20 pm/V for 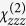 35 as compared to 188 pm/V for 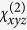 in GaAs^36^). The second order polarization is polarized along the *z* axis, i.e. no emission in the vertical direction, and we only collect a very limited fraction of the emission that is scattered toward the surface, the preferred radiation losses being in the layer plane. An integrated optimized scheme would require to engineer coupling waveguides for both pump and harmonic TM-polarized modes^37^. In forthcoming photonic circuits, we will develop architectures where the in-plane coupling of light between microdisk and waveguide is optimized as well as inverted tapers with an optimized coupling efficiency at the harmonic wavelength. The third limiting factor is the spatial overlap between modes with different radial orders. We have calculated the absolute conversion efficiency following the formalism presented in ref. 31. The conversion efficiency is proportional to the square magnitude of the 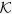 coefficient given below times the square of the free spectral range of the second harmonic. For TM-pump and TM-harmonic, the 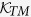 coefficient can be written as


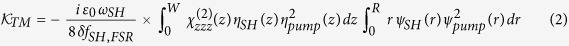


with the electric field written in cylindrical coordinates 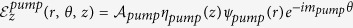. W is the disk thickness and R the disk radius. 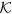 is related to *K*_*SH*_ in equation (1) by 
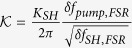
. The 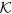 coefficient in (W s)^−1/2^ is 245 for the gallium nitride microdisks investigated in this work as compared to 6670 calculated in ref. 31 for a GaAs microdisk. The calculated conversion efficiency for the investigated structure is thus 1.4 × 10^−4^ mW^−1^. We mainly attribute the difference with the experimental value to the poor collection efficiency for the microdisk harmonic emission that preferentially radiates in the layer plane.

There are several options to increase the value of the 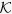 coefficient for the gallium nitride microdisks. The first one would be to use a TE-TM conversion scheme with modes with the same radial number. This could be achieved with thin layers (170 nm) and a disk diameter of 18 *μ*m. The 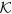 coefficient is in this case 1625 for phase matching between TE(0, 0, 47) and TM(0, 0, 94) whispering gallery modes. The second option is to take advantage of a specific property of III-N materials. The sign of the second order nonlinear susceptibility can be changed depending on the polarity of the GaN layer, either N or Ga by inserting Mg doping during the growth^38^. With this vertical inversion, phase matching can thus be considered between modes whose vertical number differs only by one, instead of 2 for the radial order in the present experiments. The polarity inversion allows to increase the modal overlap in *z* direction as given by equation (2). For a 6 *μ*m disk diameter and 560 nm thickness, the 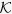 coefficient can reach 665 for conversion between TE(0, 0, 19) and TM(1, 0, 38) modes along with a large free spectral range as the disk diameter is only 6 *μ*m. In the case of TE(0, 0, 47)-TM(0, 0, 94) conversion, the calculated efficiency in mW^−1^ for a 1 mW pump and Q factors of 10000 is expected to be around 0.015%. For the TE(0, 0, 19)-TM(1, 0, 38) conversion scheme, the efficiency reaches 0.08% as compared to 1.2% calculated for a GaAs microdisk^31^. The efficiency for gallium nitride microdisks is below the one of gallium arsenide microdisks but the III-nitride materials offer a much larger transparency window with the possibility of conversion between telecom wavelengths and visible spectral range. Higher efficiencies can be expected if higher quality factors are experimentally obtained^22^.

## Conclusion

In conclusion, we have demonstrated phase-matched second harmonic generation in gallium nitride microdisks on silicon. The nonlinear experiments were performed with a sub-mW continuous wave pump in the near-infrared. The microdisks are embedded in photonic circuits and light is coupled through suspended bus waveguides, a noticeable difference as compared to previous reports using elongated fibers. This architecture is more robust, has better reproducibility and paves the way to an integrated architecture for gallium nitride microresonators. The phase matching was demonstrated by an original method based on the systematic variation of one microdisk parameter, its diameter varied by steps of 8 nm, i.e three orders of magnitude smaller than the disk diameter. With a series of microdisks, it was possible to observe the achievement of double resonance conditions between modes satisfying the conservation of wavevectors. For a detuning smaller than the full width at half maximum of the pump mode, there is an enhancement of the conversion efficiency due to both pump and harmonic resonances that demonstrates the phase matching achievement. We have proposed strategies to increase the conversion efficiency by using the peculiar features of III-nitride materials and inverting the polarity of the layer in the vertical direction. The proposed architecture is also compatible with growth of the III-nitride on silicon-on-insulator substrates. The demonstration of phase matching conversion is an important step to demonstrate the full potential of III-nitride optical circuits on silicon. This architecture could be exploited for harmonic conversion with intrinsic III-nitride emitters as active layers or to perform visible to near-infrared spontaneous down-conversion for quantum optics experiments^39^,^40^.

## Methods

The crystalline III-nitride layers were grown by molecular beam epitaxy on Si(111) substrates. An AlN buffer layer is first grown on silicon^8^. Its thickness (245 nm) is adjusted in order to reach an optimum structural quality and to introduce enough compressive strain in the following epitaxial GaN layer (497 nm thick). This compressive strain partially compensates the tensile strain appearing during cooling due to the difference of thermal expansion coefficients between the III-nitrides and the silicon substrate. Finally, strain compensation allows one to dispose of suspended planar materials after underetching on tens of micrometers length scales. We have chosen to work with TM modes both for the pump and the second harmonic since it allows us to use the highest nonlinear coefficient 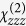 for the second harmonic generation. The thickness of the epitaxial layer was thus adjusted so that the phase matching condition around 1550 nm could be realised for sufficiently large microdisks to avoid radiation losses and to get better mode confinement which makes modes less sensitive to the fabrication imperfections.

The photonic circuit consists of microdisks coupled to free-standing waveguides suspended by nanotethers. The microdisks are mushroom-type with a silicon pedestal. The coupling between microdisks and waveguides is controlled by the gap distance between both. The advantage of the coupling scheme is its mechanical stability and reproducibility as compared to coupling with an elongated fiber at the proximity of the microdisks^23^,^34^ or even in contact with microdisks^4^. The air gap distance was chosen in order to be at the critical coupling, i.e. for an optimum transfer of light from the waveguide to the microdisk. Light is injected by lensed fibers through inverted tapers. The global length of the structure is 500 *μ*m. Fabrication of the structures is achieved by a combination of electron beam lithography and inductively coupled dry etching. Selective under-etching between III-nitrides and silicon is performed with XeF_2_ gas. The dark contrast in the top part of Fig. 1(b) indicates that the structures lye on silicon while the light contrast corresponds to free-standing structures in air. One clearly sees the silicon pedestal of the microdisk. For phase matching experiments, a series of microdisks were fabricated with a variation of 8 nm in the disk diameter. This high precision on the disk diameter is obtained through dry etching and is an advantage as compared to the precision achieved with wet etching^34^.

The harmonic experiments were performed with a continuous wave laser source in the telecom band (1500–1630 nm) delivering up to 10 dBm at the laser output. The transmission losses are around 0.5 dB for polarization control, 5 dB per taper around 1510 nm and 4 dB due to the scattering of the nanotethers between taper and microdisk. 10 dBm of laser power thus corresponds to around 1.1 mW in the bus waveguide close to a microdisk. The harmonic was collected perpendicularly to the layer plane with a high numerical aperture objective (0.9). The harmonic emission was selected with bandpass filters and was imaged with an electron multiplying charge coupled camera.

## Additional Information

**How to cite this article**: Roland, I. *et al*. Phase-matched second harmonic generation with on-chip GaN-on-Si microdisks. *Sci. Rep.* **6**, 34191; doi: 10.1038/srep34191 (2016).

## Supplementary Material

Supplementary Information
