# Supplementary material for: Phase-matched second harmonic generation with on-chip GaN-on-Si microdisks
Source: Sci Rep. 2016 Sep 30;6:34191. doi: 10.1038/srep34191 (PMC5043238; doi:10.1038/srep34191)
Supplement: Supplementary Information [file srep34191-s1.pdf]

Phase-matched second harmonic generation with on-chip GaN-on-Si microdisks: Supplementary

I. Roland[1], M. Gromovyi[2], Y. Zeng[1], M. El Kurdi[1], S. Sauvage[1], C. Brimont[3], T. Guillet[3], B. Gayral[4,5], F. Semond[2], J.Y. Duboz[2], M. de Micheli[6], X. Checoury[1], and P. Boucaud[1,*]

[1]Centre de Nanosciences et de Nanotechnologies, CNRS, Univ. Paris-Sud, Université Paris-Saclay, Bâtiment 220, Rue André Ampère, F-91405 Orsay, France
[2]CRHEA-CNRS, Rue Bernard Grégory, F-06560 Valbonne, France
[3]Laboratoire Charles Coulomb (L2C), UMR 5221, CNRS-Université de Montpellier, F-34905 Montpellier, France
[4]Univ. Grenoble Alpes, F-38000 Grenoble, France
[5]CEA, INAC-PHELIQS, Nanophysique et semiconducteurs group, F-38000 Grenoble, France
[6]Laboratoire de Physique de la Matière Condensée, UMR CNRS 7336, Université de Nice-Sophia Antipolis, 06108 Nice, France
[*]Corresponding author: philippe.boucaud@ief.u-psud.fr

ABSTRACT

Supplementary material for manuscript "Phase-matched second harmonic generation with on-chip GaN-on-Si microdisks"

1 Phase matching between TM(0,0,28) and TM(0,2,56) modes

The figure S1 shows the resonance wavelength of the TM(0,0,28) and TM(0,2,56) modes as a function of the disk diameter. Phase matching is obtained for a disk diameter of 7.99 μm. We attribute the difference of diameter with the experimental value to the uncertainty on the refractive indices at the harmonic wavelength and to the uncertainty on the disk diameter as a small tilt in the vertical sidewall is present in the processed structures. A variation of the refractive index by 0.01047 for the harmonic wavelength would predict phase matching at 7.6 μm as observed experimentally.

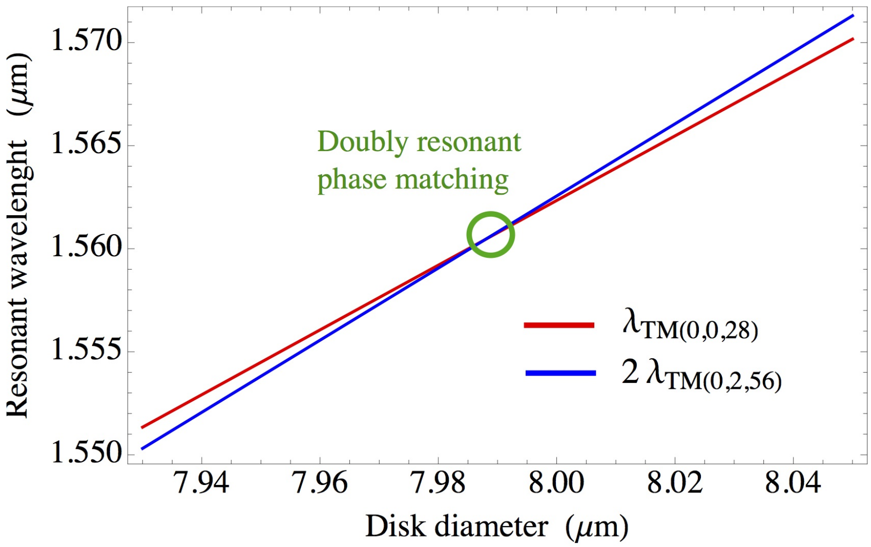

Figure S 1. Calculated dependence of the resonance wavelength of the TM(0,0,28) mode and twice the wavelength of the TM(0,2,56) modes as a function of the disk diameter.

2 Harmonic generation measured on a large spectral range

The figure S2 corresponds to the measurement of the second harmonic signal on a large spectral range for the disk diameter of 7616 nm. The second harmonic efficiency has been normalized to one when the pump is in resonance with the TM(0,0,28) mode. Single-resonance second harmonic signal can be observed when the pump is in resonance with other whispering gallery modes in the near infrared, like the TM(0,0,27) mode.

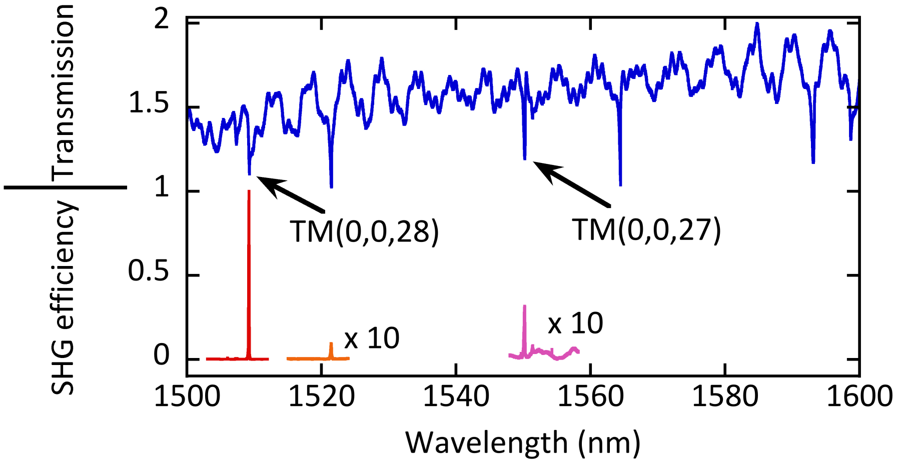

Figure S 2. Figure showing the SHG efficiency obtained with the excitation of different modes. The upper part of the curve corresponds to the transmission measurement. The lower part of the curve corresponds to the SHG efficiency (SHG signal divided by the square of the coupled power). The SHG efficiency has been normalized to 1 for the maximum obtained with the TM(0,0,28) mode. The efficiency is much smaller with the other modes (the vertical scale has been multiplied by a factor of 10). The measurement is performed with the 7616 nm diameter disk for which phase matching is obtained with the TM(0,0,28) mode. The amplitudes of the oscillation on the transmission measurement can vary depending on the experimental setting.

3 Modeling of second harmonic signal

The figure S3 shows some examples of modeling of the SHG signal following Formula 1. The amplitude varies for each curve as well as the vertical scale. One clearly observes the effect of the double resonance when the harmonic is also resonant with a whispering gallery mode. The modeling does not account for the splitting of the harmonic mode.

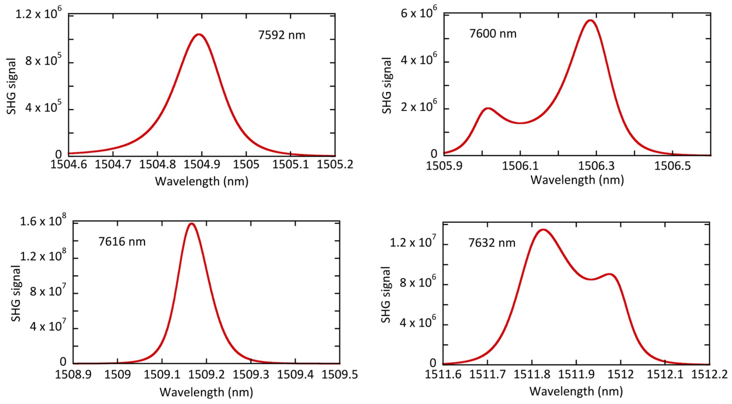

Figure S 3. Examples of modeling of the results presented in Fig. 3 following formula 1. Note that the vertical scale differs for each curve. The amplitude of the maximum SHG signal is used to draw Figure 4. One can directly see the impact of the double resonance (diameter 7616 nm) where the harmonic signal is significantly higher. The modeling does not account for the splitting between clockwise and counter-clockwise mode for the harmonic whispering gallery mode. The disk diameter is indicated on each curve.

4 Susceptibility dispersion

The ordinary and extraordinary relative susceptibility dispersions used for the III-nitride materials in the modeling are obtained following Ref.[1] and Ref.[2]. The wavelength is expressed in μm.

AlN

$$\varepsilon_o = 1 + \frac{3.14768\,\lambda^2}{\lambda^2 - 0.018249}$$
$$\varepsilon_e = 1 + \frac{3.33537\,\lambda^2}{\lambda^2 - 0.019368}$$

GaN

$$\varepsilon_o = 1 + \frac{0.213\,\lambda^2}{\lambda^2 - 0.1225} + \frac{3.988\,\lambda^2}{\lambda^2 - 0.02341}$$
$$\varepsilon_e = 1 + \frac{0.118\,\lambda^2}{\lambda^2 - 0.1225} + \frac{4.201\,\lambda^2}{\lambda^2 - 0.03115}$$

References

1. Ozgur, U., Webb-Wood, G., Everitt, H. O., Yun, F. & Morkoç, H. Systematic measurement of $Al_xGa_{1-x}N$ refractive indices. *Applied Physics Letters* **79**, 4103–4105 (2001). URL http://scitation.aip.org/content/aip/journal/apl/79/25/10.1063/1.1426270.

2. Pezzagna, S., Brault, J., Leroux, M., Massies, J. & de Micheli, M. Refractive indices and elasto-optic coefficients of GaN studied by optical waveguiding. *Journal of Applied Physics* **103**, 123112 (2008). URL http://scitation.aip.org/content/aip/journal/jap/103/12/10.1063/1.2947598.
